# Oxygen and Carbon Isotope Variations in a Modern Rodent Community – Implications for Palaeoenvironmental Reconstructions

**DOI:** 10.1371/journal.pone.0049531

**Published:** 2012-11-19

**Authors:** Alexander Gehler, Thomas Tütken, Andreas Pack

**Affiliations:** 1 Georg-August-Universität, Geowissenschaftliches Zentrum, Abteilung Isotopengeologie, Göttingen, Deutschland; 2 Rheinische Friedrich-Wilhelms-Universität, Steinmann-Institut für Geologie, Mineralogie und Paläontologie, Emmy Noether-Gruppe Knochengeochemie, Bonn, Deutschland; Raymond M. Alf Museum of Paleontology, United States of America

## Abstract

**Background:**

The oxygen (δ^18^O) and carbon (δ^13^C) isotope compositions of bioapatite from skeletal remains of fossil mammals are well-established proxies for the reconstruction of palaeoenvironmental and palaeoclimatic conditions. Stable isotope studies of modern analogues are an important prerequisite for such reconstructions from fossil mammal remains. While numerous studies have investigated modern large- and medium-sized mammals, comparable studies are rare for small mammals. Due to their high abundance in terrestrial ecosystems, short life spans and small habitat size, small mammals are good recorders of local environments.

**Methodology/Findings:**

The δ^18^O and δ^13^C values of teeth and bones of seven sympatric modern rodent species collected from owl pellets at a single locality were measured, and the inter-specific, intra-specific and intra-individual variations were evaluated. Minimum sample sizes to obtain reproducible population δ^18^O means within one standard deviation were determined. These parameters are comparable to existing data from large mammals. Additionally, the fractionation between coexisting carbonate (δ^18^O_CO3_) and phosphate (δ^18^O_PO4_) in rodent bioapatite was determined, and δ^18^O values were compared to existing calibration equations between the δ^18^O of rodent bioapatite and local surface water (δ^18^O_LW_). Specific calibration equations between δ^18^O_PO4_ and δ^18^O_LW_ may be applicable on a taxonomic level higher than the species. However, a significant bias can occur when bone-based equations are applied to tooth-data and vice versa, which is due to differences in skeletal tissue formation times. δ^13^C values reflect the rodents’ diet and agree well with field observations of their nutritional behaviour.

**Conclusions/Significance:**

Rodents have a high potential for the reconstruction of palaeoenvironmental conditions by means of bioapatite δ^18^O and δ^13^C analysis. No significant disadvantages compared to larger mammals were observed. However, for refined palaeoenvironmental reconstructions a better understanding of stable isotope signatures in modern analogous communities and potential biases due to seasonality effects, population dynamics and tissue formation rates is necessary.

## Introduction

Stable isotope compositions of mammalian bioapatite are widely used proxies in palaeoenvironmental and palaeodietary studies. Starting with pioneering research on carbon [1] and oxygen isotopes [2]–[5] in the 1970s and 1980s, stable isotope analysis of bioapatite from fossil mammals rapidly became an established method for the reconstruction of palaeoclimate and palaeodiet.

The oxygen isotope composition of bioapatite from terrestrial mammals can be used to infer air temperature, climate seasonality, relative humidity or aridity of palaeoenvironments as well as mobility, birth seasonality and drinking behaviour of specific mammal taxa (e.g. [6] and references therein; [7]–[11]). Carbon isotopes can be used to infer the palaeovegetation, vegetation cover, dietary strategies, resource partitioning and habitat use (e.g. [6] and references therein; [8], [9], [12]–[15]).

For various reasons, especially to allow easier sampling and acquisition of sufficient sample material, most studies have focused predominantly on the isotopic analysis of skeletal remains from large mammals. New and improved mass spectrometric techniques allow oxygen and carbon isotope analysis of (sub)milligram sample amounts of bioapatite, bringing small mammal taxa such as small rodents (with a body mass below 1 kg) into the focus of interest.

So far only a few oxygen and/or carbon isotope studies are based exclusively or partly on fossil small rodents [8], [16]–[27]. However, for an improved understanding of oxygen and carbon isotope compositions in bioapatite of fossil small rodents, communities of modern analogues have to be studied extensively in order to investigate inter- and intra-specific variabilities. This has been done repeatedly for selected large mammals (e.g., [28]–[35]) but no comparable study has been conducted on small mammals so far. It is the aim of the present study to investigate such variations for small mammals. Such studies are fundamental in order to evaluate the number of individuals needed for statistically significant results and to ascertain whether species-specific calibrations or calibrations on a higher taxonomic level are suitable for the reconstruction of the oxygen isotope composition of local surface water. Furthermore, the intra-individual variability between bones and permanently growing teeth as well as the intra-jaw variations need to be determined in order to investigate if these skeletal tissues record comparable isotope signatures or are seasonally biased.

We analysed the oxygen (δ^18^O) and carbon isotope (δ^13^C) composition (for definitions see Materials and Methods section) of bones and teeth from seven rodent species in multiple individuals. The samples derive from owl pellets of a locality in northwestern Germany, which were accumulated over a 4-year period (1991–1995). Inter-specific, intra-specific, and intra-jaw variations were investigated, as well as variations between teeth and bones from the same individuals. These data were compared to published stable isotope data for large mammals.

Additionally, oxygen isotope analyses of coexisting carbonate (δ^18^O_CO3_) and phosphate (δ^18^O_PO4_) in rodent bioapatite were conducted on water voles (*Arvicola terrestris*). In order to determine if it is possible to make a correct estimation of the δ^18^O_LW_ from the δ^18^O_CO3_ values, the data from the other species were converted to δ^18^O_PO4_ equivalent values by using the average offset between δ^18^O_CO3_ and δ^18^O_PO4_ (Δ^18^O_CO3-PO4_) obtained for *A*. *terrestris*. Then the data were compared to the three published δ^18^O_PO4_-δ^18^O_H2O_ calibration equations determined for different extant rodent taxa in previous studies [4], [16], [36].

With the present study, the authors aim to provide a basic contribution for the enhancement of palaeoenvironmental interpretations of oxygen and carbon isotope data obtained from fossil rodents.

### General Considerations

#### Oxygen isotopes

The oxygen isotope composition of mammalian bioapatite is determined by that of body water, which in turn is controlled by the oxygen isotope compositions of the different oxygen input sources (drinking water, food water, air oxygen, organic food compounds and water vapour in air) and oxygen output fluxes (exhaled CO_2_, liquid water in sweat, urine and feces as well as orally, nasally and transcutaneously released water vapour) [(e.g., [4], [33], [37]). The oxygen isotope composition of mammalian body water is linearly related to that of their drinking water (i.e., local surface water) for those mammals with an obligate drinking behaviour (e.g., [2], [38]). Thus, empirical specific calibration equations relating δ^18^O_PO4/CO3_ and δ^18^O_LW_ can be developed. Because the oxygen isotope composition of local surface water varies with air temperature and also with the amount of local precipitation and evapotranspiration (e.g., [39]–[42]), (palaeo-)climatic conditions can be inferred from the oxygen isotope composition of the skeletal remains of fossil mammals (e.g., [9] and references therein).

In bioapatite, oxygen is present in the phosphate, carbonate and the hydroxyl groups [6]. The average offset between δ^18^O_PO4_ and δ^18^O_CO3_ in skeletal apatite of different mammal taxa is around 9‰. This offset ranges from 7.5 to 11.4‰ in the previously investigated modern taxa [43]–[49]. Both, δ^18^O_PO4_ and δ^18^O_CO3_, can be used to track the oxygen isotope composition of ingested drinking water. For the offset between δ^18^O_PO4_ and oxygen bound in the hydroxyl group, only a single calculated value of −16.6‰ is known [50].

#### Carbon isotopes

The carbon isotope composition of mammalian bioapatite is controlled by that of ingested food (i.e., plant material in herbivores) [1] and can therefore be used to investigate dietary preferences. Furthermore, the knowledge of the carbon isotope composition of ingested plant material can mirror specific habitats, type of vegetation cover (i.e., C_3_ versus C_4_ plants) and density, as well as ecological niches,feeding behaviour and seasonal changes of diet (e.g., [9] and references therein).

Significant differences in the carbon isotope composition of plants are induced by different carbon fixation strategies in plants, i.e., the C_3_, C_4_ or CAM (Crassulacean Acid Metabolism) photosynthetic pathways (e.g. [51]–[53]). C_3_ plants, which represent about 95% of all terrestrial plant species (e.g., [54]), have typical δ^13^C values between -36 and −22‰ with an average value of −27‰, whereas the δ^13^C values of C_4_ plants (mostly warm season grasses and sedges) have −15 to −10‰, with an average value of −13‰ (e.g. [51], [52], [55], [56]). CAM plants have highly variable δ^13^C values within and between specific taxa, overlapping with both C_3_ and C_4_ plants (e.g., [55], [57], [58]). However, CAM plants represent only about 4% of all terrestrial plant species [54] and are mostly succulents [59], playing a minor role in the nutrition of most herbivorous mammals. Thus, a distinction between browsing and grazing herbivores based on bioapatite δ^13^C values is possible, as shown by numerous palaeodietary studies (e.g. [60]–[65]). However, this approach is limited to ecosystems with coexisting C_3_ and C_4_ plants; the latter were not globally abundant until the late Miocene [66].

Even in a pure C_3_ ecosystem, significant differences in the carbon isotopic composition of plants are caused by varying light-, nutrient-, water-, CO_2_- and temperature-settings (e.g., [51], [67]). Subcanopy plants in humid, shaded environments assimilating CO_2_, depleted in ^13^C by soil respiration, have very low δ^13^C values ranging from −36 to −32‰ [12], [51], [68]–[71]. C_3_ plants in arid, open environments have the highest δ^13^C values, up to −21‰ [51], [52], [72]. Those variations are also visible in the carbon isotope composition of bioapatite from modern and fossil mammals living in C_3_-environments and reflect differences in habitat use and/or resource partitioning (e.g., [9], [12], [13], [15], [73]–[78]). Terrestrial C_3_ plant ecosystems date back to the Palaeozoic and are typical for most parts of the modern northern hemisphere (e.g., [79]).

The carbon isotope composition of mammalian bioapatite is enriched by several per mil compared to the respective diet. Reported enrichment factor values range from 9 to 15‰, depending on the digestive physiology of the investigated taxa (e.g., [80] and references therein).

#### Stable carbon and oxygen isotopes from bioapatite of fossil small rodents and case studies on modern material

Mammals evolved in the Late Triassic and retained relatively small body size until their radiation after the Cretaceous-Palaeogene transition [81]. Rodents first appear in the Palaeogene fossil record, and fossil rodent remains are widespread in Cenozoic terrestrial deposits, often in high numbers, accumulated predominantly by ancient avian predators. As reviewed by Grimes et al. [82], one of the main advantages of stable isotope studies on small mammals relative to large taxa is the higher abundance of small mammals in the fossil record, which enhances their availability. Furthermore, small mammals display a rapid evolution of their morphology, and hence small mammal remains are often index fossils for Cenozoic strata that enable a good biostratigraphic resolution. Additionally, most small mammals occupy a very restricted habitat, lacking long distance migratory behaviour, thus reflecting local palaeoenvironmental conditions more precisely than large mammals. Possible disadvantages compared to large mammals concerning the oxygen isotope composition may be a smaller proportion of drinking water in the overall oxygen intake, a more variable physiology (i.e., body temperature), and a possible stronger susceptibility to diagenetic alteration due to smaller, more fragile skeletal elements [82].

Specific calibration equations between δ^18^O_PO4_ and δ^18^O_LW_ have been developed in the past for laboratory rats [4], wild murids [36], [38] and wild arvicolids [16], [36]. During the last decade, the oxygen isotope composition of bioapatite of fossil small rodents has been used in an increasing number of studies, targeting Palaeogene [20]–[22], [27], Neogene [8], [26], [27] and Pleistocene [16]–[19] climate change. Carbon isotope records of fossil small rodents also were used to reconstruct vegetational change and palaeodiets in the Cenozoic [18], [19], [23]–[25].

To date, very little is known about the inter- and intra-population variability between individuals from the same locality, as well as about variations between different teeth and bone material within a single individual. Only one study [83] has investigated intra-population and intra-jaw variations (δ^18^O_PO4_) in small rodents based on a small number of samples of fat dormice (*Glis glis*, n = 3) from Great Missenden (Buckinghamshire, UK) and wood mice (*Apodemus sylvaticus*, n = 5) from Dungeness (Kent, UK). From these results, Lindars et al. [83] concluded that a quantity of>5 different post-weaning teeth should be used for isotope palaeo-thermometry. Further intra-population δ^18^O_PO4_ data of multiple bioapatite analyses from small rodents (*Apodemus flavicollis*, *Apodemus sylvaticus*, *Arvicola terrestris*, *Microtus arvalis* and *Pitymus* sp.) are presented in a small quantity (n = 3–8) by D’Angela and Longinelli [38] and Longinelli et al. [36], but no data for distinct sympatric species from the same locality are available so far.

## Materials and Methods

### Material

**Figure 1:**
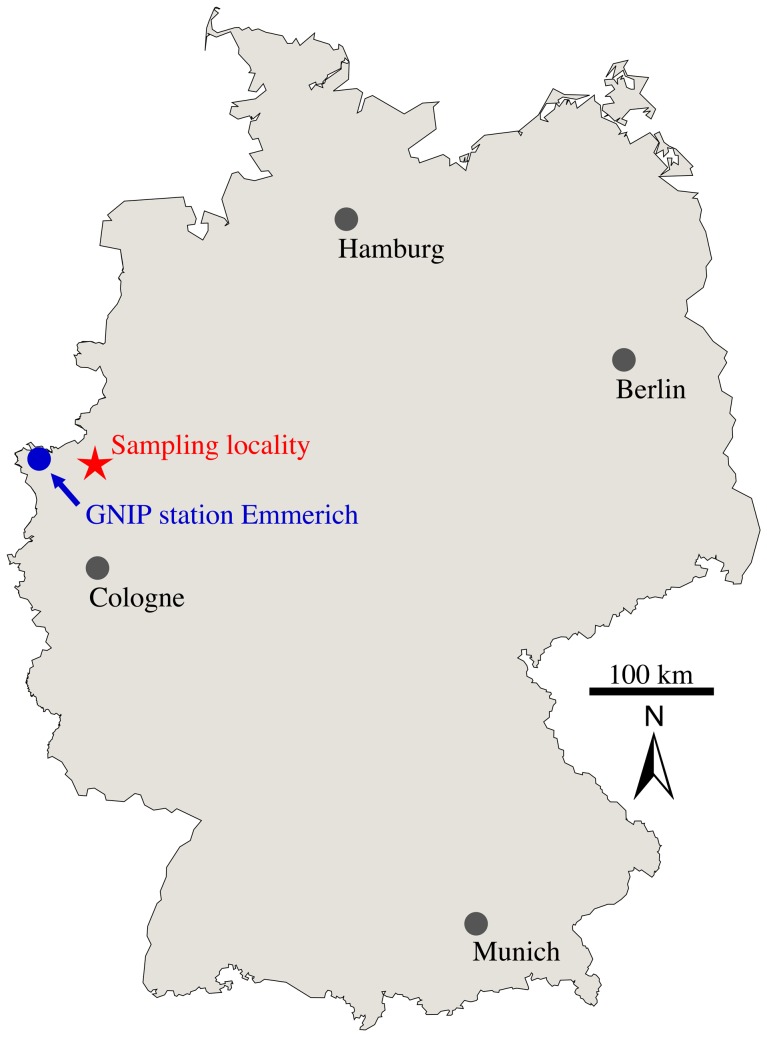
Map showing the sampling locality Rhader Wiesen near Dorsten, North Rhine-Westphalia, Germany (red star) and the closest IAEA-GNIP station to it (blue dot).

**Figure 2:**
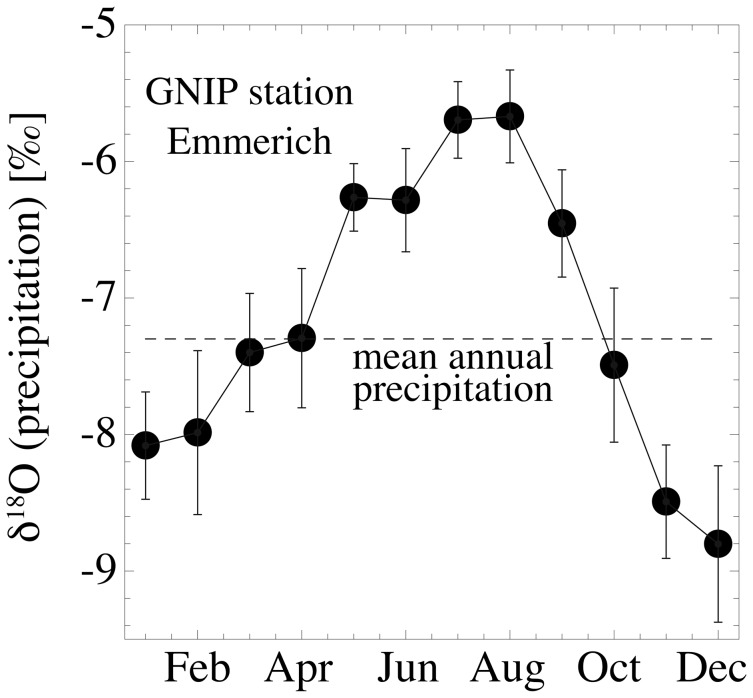
Mean long-term monthly δ^18^O record (1980–2005) of local precipitation from the IAEA-GNIP station Emmerich.

The samples originate from fresh barn owl (*Tyto alba*) pellets accumulated over a maximum of four years, collected in April 1995 at “Hof Gülker”, located in the nature reserve Rhader Wiesen, about 9 km north of Dorsten, North Rhine-Westphalia, Germany (Fig. 1) and are part of the original material from Bülow [84]. Long term mean annual local temperatures in this region (IAEA-GNIP station Emmerich) average 10°C, and the mean annual precipitation is 744 mm. The long term weighted mean of the monthly δ^18^O record of local precipitation between 1980 and 2005 is −7.3‰. Considering only the period from April 1991 to April 1995 of owl pellet deposition, and hence the most likely time interval when the analysed rodents lived and mineralised their bones and teeth, the respective δ^18^O value is −7.7. This is very close to the long term record [85]. The seasonal change in monthly precipitation of the sampling area is illustrated in Fig. 2.

The rodent skeletal material belongs to the four arvicolid species *Arvicola terrestris*, *Myodes glareolus*, *Microtus agrestis* and *Microtus arvalis* as well as to the three murid species *Apodemus sylvaticus*, *Mus musculus*, and *Rattus norvegicus*. From the latter species, the owl pellets contained only juvenile individuals. The analysed teeth were permanent growing (arvicolid and murid incisors and most arvicolid molars), thus reflecting the last four to twelve weeks in the life of the respective individual prior to death [86], [87]. Bone material comprises stable isotope compositions over a longer time period, approaching the life span of the individual [e.g. 88], which in the present case is the time until predation by barn owls. None of the analysed taxa hibernate.

The isotope signatures of the skeletal tissue samples are likely to be seasonally biased, because of the different tissue formation and turnover periods and due to the seasonal population dynamics of the rodents and their predators. Population sizes of arvicolids and murids in temperate regions typically increase after a minimum at the end of the winter with the first breed in spring and reach a maximum (partly up to more than one order of magnitude larger than the early spring population) in late summer (e.g., [89]–[91]). Avian predator populations, responsible for pellet accumulations, are positively correlated to populations of their small mammal prey (e.g., [92]–[95]), causing a warm-season biased pellet deposition in modern ecosystems and probably in fossil ecosystems as well. Analogous conditions for modern and fossil samples are an important prerequisite for the development of δ^18^O_PO4/CO3_-δ^18^O_LW_ calibration equations and their application for palaeoclimate reconstructions [16].

No specific permits were required for the described field studies.

### Methods

#### Oxygen and carbon isotope analyses of the carbonate in the bioapatite (δ^18^O_CO3_, δ^13^C)

Only teeth from taxonomically identified jaw specimens were used for stable isotope analysis. Teeth from each species, both upper incisors (in *Arvicola terrestris* and *Rattus norvegicus* only the left upper incisor) of ten individuals, were extracted from the jaw and inspected to ensure that no signs of digestive etching were present. Further δ^18^O_CO3_ and δ^13^C analyses were conducted on jaw bone material (derived from the zygomatic region) of five of the ten individuals of each species. In *Arvicola terrestris*, the left upper molars (M1–M3) of the ten specimens were also analysed.

Bulk tooth and bone material was crushed and ground to a fine powder using an agate mortar and pestle. The chemical pretreatment procedure to remove organic matter and adherent carbonates followed Koch et al. [96]. Approximately 10 mg of sample powder were soaked with 30% H_2_O_2_ (0.1 ml mg^−1^) for 24 h, rinsed five times with millipore water, soaked for another 24 h with an acetic acid - calcium acetate buffer solution (1 M, pH = 5, 0.05 ml mg^−1^) and rinsed again five times with millipore water, followed by drying overnight at 50°C. Typically, 1 to 1.3 mg of pretreated sample powder were reacted for 15 min. with 100% H_3_PO_4_ at 70°C in a Thermo Scientific KIEL IV automated carbonate device. Released CO_2_ was measured in dual inlet mode with a Finnigan Delta plus isotope ratio gas mass spectrometer at the stable isotope laboratory of the Geoscience Center at the University of Göttingen. The measured isotope compositions were normalised to the NBS 19 calcite standard, measured in the same runs together with the bioapatite samples. Data are reported in the δ-notation in per mil (‰), relative to the international isotope reference standards Vienna Standard Mean Ocean Water (VSMOW) for δ^18^O and Vienna Pee Dee Belemnite (VPDB) for δ^13^C [97].


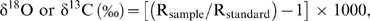


where R_sample_ and R_standard_ are the ^18^O/^16^O and ^13^C/^12^C ratios in sample and standard, respectively.

The analytical precision for the NBS 19 was ±0.1‰ (1σ) in δ^18^O and ±0.04‰ (1σ) in δ^13^C (n = 232, analysed between November 2010 and February 2012). For the NBS 120c Florida phosphate rock standard (pretreated as described above), we obtained a δ^18^O value of 30.0±0.2‰ (1σ) and a δ^13^C value of −6.4±0.04‰ (1σ) (n = 10). Our internal bioapatite standard AG-Lox (African elephant enamel) had a δ^18^O value of 30.0±0.08‰ (1σ) and a δ^13^C value of -12.0±0.03‰ (1σ) (n = 11). For inter-laboratory comparison, material of our internal AG-Lox enamel standard will be supplied upon request by the authors.

#### Oxygen analyses of the phosphate (δ^18^O_PO4_)

Analyses of the phosphate moiety of the bioapatite (δ^18^O_PO4_) were conducted on the incisors, M1 and bone material from five of the *Arvicola terrestris* specimens.

Ag_3_PO_4_ was precipitated from about 4 mg of the pretreated sample powder (see section above), using a method slightly modified after Dettmann et al. [98] and described in detail by Tütken et al. [8]. For dissolution of the samples, 0.8 ml of HF (2 M) were added, and the sample vials were put on a vibrating table for 12 h. This was followed by centrifugation and transfer to new vials of the supernatant sample solutions, leaving behind the CaF solid residue. After neutralising the HF solution with NH_4_OH (25%) in the presence of bromothymol blue as pH-indicator, Ag_3_PO_4_ was precipitated rapidly by adding 0.8 ml of 2 M silver nitrate (AgNO_3_) solution. After settling of the small Ag_3_PO_4_ crystals and centrifugation, the supernatant solution was pipetted off and the Ag_3_PO_4_ was rinsed twice with 1.8 ml millipore water. The Ag_3_PO_4_ was then dried overnight in an oven at 50°C.

Ag_3_PO_4_ aliquots of 0.5 mg were placed into silver capsules and analysed in triplicate by means of high temperature reduction Finnigan TC-EA coupled via a Conflo III to a Finnigan Delta Plus XL GC-IRMS, according to the method of Vennemann et al. [99].

### Statistical Methods

The statistical analyses were performed using Wolfram Mathematica 7.0 and 8.0. To evaluate if the differences in the mean isotope values among multiple taxa are statistically significant, one-factor analyses of variance (ANOVA) were conducted, followed by posthoc-tests (Tukey) for pairwise comparisons. In one case, the assumptions of the parametric ANOVA were violated (non-normal distribution), therefore we additionally performed a Kruskal-Wallis non-parametric ANOVA that was not in disagreement with the parametric ANOVA results.

Minimum sample sizes to represent the population mean in δ^18^O by 95% degree of confidence within ±1σ were determined by a standard bootstrapping approach according to the method presented by Fox-Dobbs et al. [100]. From the bulk incisor data sets of the seven analysed species (n = 10 each), 1,000 randomly selected subsamples (n_sub_) were generated for every n_sub_ between 2 and n-1. Then, the proportion of subsamples within all random replicates for a given n_sub_ that range between ±1σ of the mean of the original datasets was determined, using the average value from 100 repeated evaluations. If the mean values of ≥950 out of 1000 subsamples are in a range between ±1σ of the analysed dataset, it is indicated that the respective n_sub_ is adequate to estimate the population mean at least by a 95% confidence level.

## Results

### Oxygen and Carbon Isotope Data of the Carbonate in the Bioapatite (δ^18^O_CO3_, δ^13^C)

The data, which are summarised in Table S1, include 135 individual δ^18^O_CO3_ and δ^13^C analyses of bulk incisor and molar teeth, as well as δ^18^O_CO3_ and δ^13^C values of bone material from seven modern arvicolid and murid species from a single locality in NW Germany (see Materials and Methods section).

### Oxygen Isotope Composition of the Incisors

**Figure 3:**
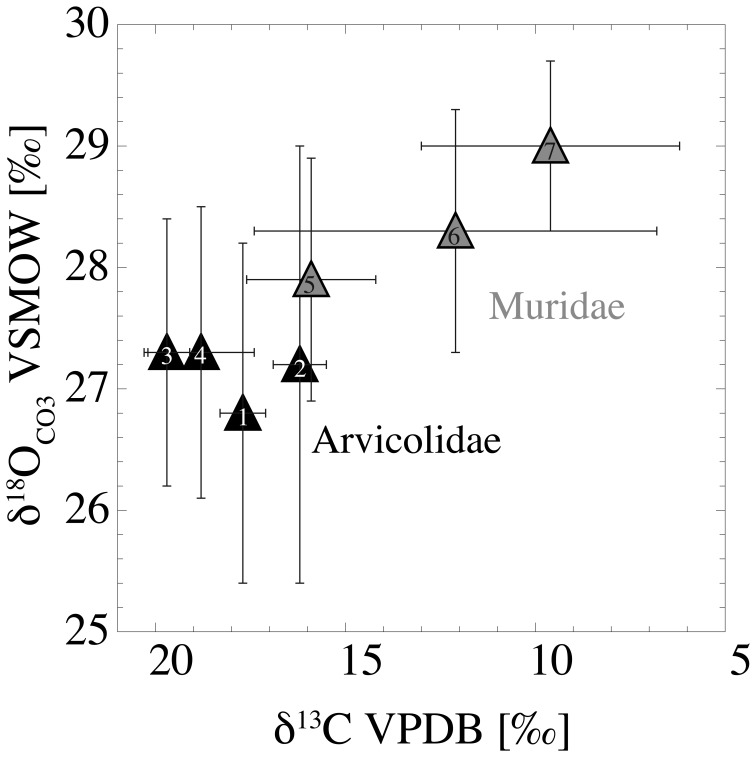
Mean δ^18^O_CO3_ and δ^13^C values of bulk incisors of the seven analysed rodent species (n = 10 in each species) with 1σ error bars. 1: *A*. *terrestris*, 2: *M*. *glareolus*, 3: *M*. *agrestis*, 4: *M*. *arvalis*, 5: *A*. *sylvaticus*, 6: *M*. *musculus*, 7: *R*. *norvegicus*.

Intra-population variations in δ^18^O_CO3_ from 2.0 to 3.9‰ have been observed in the bulk incisor samples (n = 10 of each species), with the exception of the arvicolid *M. glareolus*, which has a significantly higher range of 6.7‰. However, if the most extreme value is excluded and considered as an outlier, *M. glareolus* has an intra-population variation of 3.5‰ that falls within the range of variation of the other species (Table 1). The mean δ^18^O_CO3_ values of the arvicolid incisors are 26.8±1.4‰ (*A*. *terrestris*), 27.2±1.8‰ (*M. glareolus*), 27.3±1.1‰ (*M. agrestis*) and 27.3±1.2‰ (*M. arvalis*). The murid incisors have mean δ^18^O_CO3_ values of 27.9±1.0‰ (*A. sylvaticus*), 28.3±1.0‰ (*M. musculus*) and 29.0±0.7‰ (*R*. *norvegicus*) (Fig. 3, Table 1). Between the mean δ^18^O_CO3_ values of these species, statistically significant differences (one-way ANOVA, F = 3.638, P<0.01) were observed, pair-wise comparison revealed that only *A*. *terrestris* and *R*. *norvegicus* show significantly different values (Tukey test, P<0.01). Within arvicolids, no statistically significant differences in δ^18^O_CO3_ were detected (one-way ANOVA, F = 0.304, P = 0.82). Within murids, mean δ^18^O_CO3_ values differed significantly (one-way ANOVA, F = 3.654, P<0.05) but only between *A*. *sylvaticus* and *R*. *norvegicus* were significant differences detected by pair-wise comparisons (Tukey test, P<0.05).

**Table 1:** Mean δ^18^O_CO3_ and δ^13^C values of bulk incisors of the seven analysed rodent species (n = 10 in each species) with 1σ error and range of variation (lowest and highest analytical value of the respective species).

Species	Material	Mean δ^18^O_CO3_	Range of variation	Mean δ^13^C	Range of variation	n
		(‰ vs. VSMOW)	in δ^18^O_CO3_ values	(‰ vs. VPDB)	in δ^13^C values	
*Arvicola terrestris*	I^1^ sin.	26.8±1.4	24.9 to 28.8	−17.7±0.6	−18.5 to −16.7	10
*Myodes glareolus*	I^1^ sin. u. dex.^1^	27.2±1.8	24.7 to 28.2 (31.4)^2^	−16.2±0.7	−17.3 to −15.1	10
*Microtus agrestis*	I^1^ sin. u. dex.	27.3±1.1	26.0 to 28.8	−19.7±0.6	−20.5 to −18.6	10
*Microtus arvalis*	I^1^ sin. u. dex.	27.3±1.2	25.4 to 29.2	−18.8±1.4	−20.8 to −16.8	10
*Apodemus sylvaticus*	I^1^ sin. u. dex.	27.9±1.0	26.0 to 29.1	−15.9±1.7	−17.8 to −12.4	10
*Mus musculus*	I^1^ sin. u. dex.	28.3±1.0	26.4 to 29.7	−12.1±5.3	−17.4 to −4.7	10
*Rattus norvegicus*	I^1^ sin.	29.0±0.7	27.8 to 29.9	−9.6±3.4	−14.7 to −4.5	10

1 sin. = left, dex. = right.

2 value in brackets excluded as an outlier, see results section.

### Carbon Isotope Composition of the Incisors

The δ^13^C values of the incisor samples (n = 10 of each species) in three of the four modern arvicolids (*A. terrestris*, *M. glareolus* and *M*. *agrestis*) have very narrow intra-population variations of <2.2‰, with mean values of −17.7±0.6‰, −16.2±0.7‰ and −19.7±0.6‰, respectively. The intra-population δ^13^C variations of *M*. *arvalis* and the murid *A*. *sylvaticus* are much larger, with ranges of 4.0‰ and 5.4‰ and mean values of −18.8±1.4‰ and −15.9±1.7‰, respectively. Very large variations were observed in the two murids, *M*. *musculus* and *R*. *norvegicus*, that have an intra-population δ^13^C range of 12.7‰ and 10.2‰ with mean values of -12.1±5.3‰ and -9.6±3.4‰, respectively (Fig. 3, Table 1). Mean δ^13^C values differed significantly between the analysed species (one-way ANOVA, F = 20.632, P<0.01). By pair-wise comparison, mean δ^13^C values of two murids (*M*. *musculus* and *R*. *norvegicus*) differed significantly from those of all arvicolids as well as from *A. sylvaticus*. The mean δ^13^C value of *M*. *agrestis* showed significant differences relative to those of *A*. *sylvaticus* and *M. glareolus* (Tukey test, P<0.05). Considering the arvicolids separately, differences in the mean δ^13^C values between taxa (one-way ANOVA, F = 28.813, P<0.01) were observed. By pair-wise comparisons, significant differences between *M*. *agrestis* and *M. glareolus* themselves and with both other Arvicolidae were detected (Tukey test, P<0.05). Within murids, significant differences among taxa were present as well (one-way ANOVA, F = 7.230, P<0.01), but only *A*. *sylvaticus* and *R*. *norvegicus* show significant differences by pair-wise comparison (Tukey test, P<0.05).

### Oxygen and Carbon Isotope Compositions of the Bones

**Figure 4:**
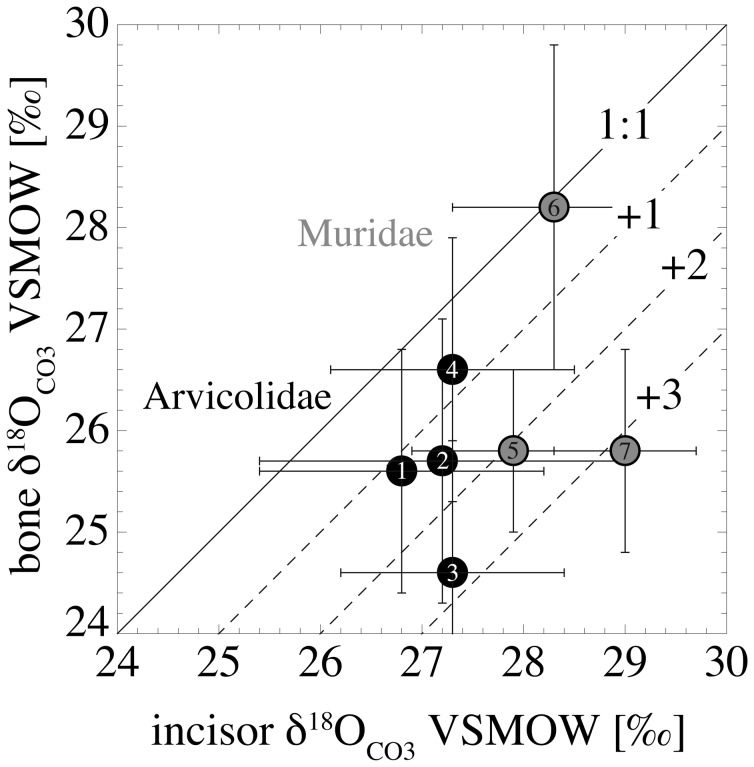
Comparison of incisor and bone δ^18^O_CO3_ values from the individuals where both tissues were analysed (n = 5 in each species). 1: *A*. *terrestris*, 2: *M*. *glareolus*, 3: *M*. *agrestis*, 4: *M*. *arvalis*, 5: *A*. *sylvaticus*, 6: *M*. *musculus*, 7: *R*. *norvegicus*. The dashed lines represent the deviation of incisor mean values from bone mean values in ‰ from the 1∶1 line (solid line).

The bone δ^18^O_CO3_ values (n = 5 of each species) deviate from the incisor values of of the same individuals by −4.2 to+1.5‰. 85% (30 out of 35 specimens) have higher δ^18^O_CO3_ values in their incisors than in their bones (Fig. 4, Table S1). Mean values are 25.6±1.2‰ (*A*. *terrestris*), 25.7±1.4‰ (*M. glareolus*), 24.6±1.3‰ (*M. agrestis*) and 26.6±1.3‰ (*M. arvalis*). The murid bones have mean δ^18^O_CO3_ values of 25.8±0.8‰ (*A. sylvaticus*), 28.2±1.6‰ (*M. musculus*) and 25.8±1.0‰ (*R*. *norvegicus*) (Table 2).

**Table 2:** Mean δ^18^O_CO3_ and δ^13^C values of bone material of the seven analysed rodent species (n = 5 in each species) with 1σ error and range of variation (lowest and highest analytical value of the respective species).

Species	Material	Mean δ^18^O_CO3_	Range of variation	Mean δ^13^C	Range of variation	n
		(‰ vs. VSMOW)	in δ^18^O_CO3_ values	(‰ vs. VPDB)	in δ^13^C values	
*Arvicola terrestris*	bone	25.6±1.2	24.6 to 27.7	−17.8±0.9	−19.0 to −16.8	5
*Myodes glareolus*	bone	25.7±1.4	24.0 to 27.3	−17.7±0.4	−18.3 to −17.3	5
*Microtus agrestis*	bone	24.6±1.3	23.1 to 26.4	−19.5±0.6	−20.0 to −18.5	5
*Microtus arvalis*	bone	26.6±1.3	24.8 to 27.9	−18.4±0.8	−19.5 to −17.6	5
*Apodemus sylvaticus*	bone	25.8±0.8	24.6 to 26.6	−16.4±1.3	−17.3 to −14.1	5
*Mus musculus*	bone	28.2±1.6	25.5 to 29.5	−11.6±5.5	−16.8 to −5.0	5
*Rattus norvegicus*	bone	25.8±1.0	24.7 to 26.7	−7.9±2.2	−10.7 to −5.8	5

The different bone-incisor offsets have standard deviations between 0.8 and 1.8‰ within each analysed species, except for the juvenile *R*. *norvegicus* specimen with a standard deviation of only 0.3‰.

**Figure 5:**
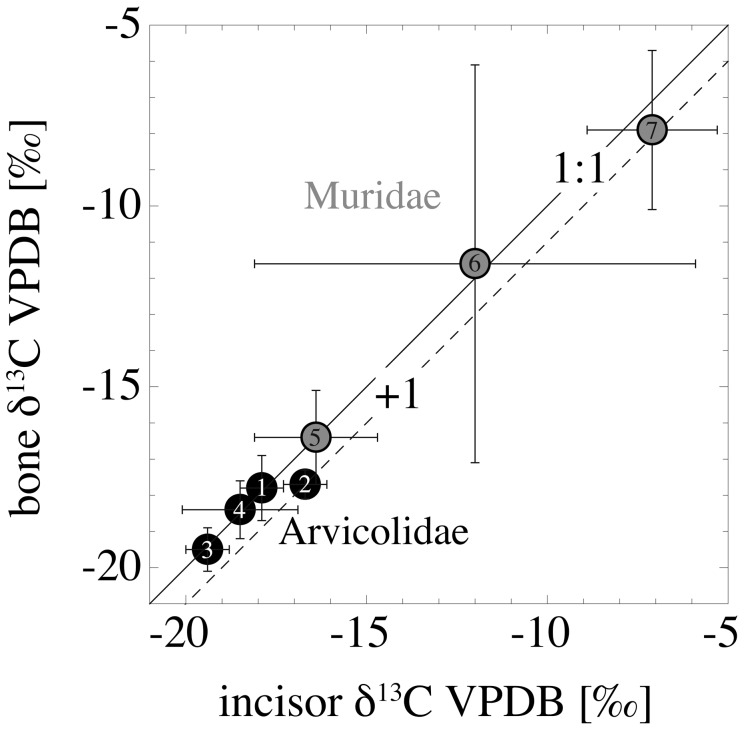
Comparison of incisor and bone δ^13^C values from the individuals where both tissues were analysed (n = 5 in each species). 1: *A*. *terrestris*, 2: *M*. *glareolus*, 3: *M*. *agrestis*, 4: *M*. *arvalis*, 5: *A*. *sylvaticus*, 6: *M*. *musculus*, 7: *R*. *norvegicus*. The dashed lines represent the deviation of incisor mean values from bone mean values in ‰ from the 1∶1 accordance (solid line).

The δ^13^C of bone material deviates by+1.9 to −1.6‰ from that of the incisors, with the exceptions of one *A*. *sylvaticus* (−2.9‰) and one *M*. *musculus* (+4.3‰). Mean values are −17.8±0.9‰ (*A*. *terrestris*), −17.7±0.4‰ (*M. glareolus*), −19.5±0.6‰ (*M. agrestis*) and −18.4±0.8‰ (*M. arvalis*). The murid bones have higher mean δ^13^C values of −16.4±1.3‰ (*A. sylvaticus*), −11.6±5.5‰ (*M. musculus*) and −7.9±2.2‰ (*R*. *norvegicus*). No systematic differences between incisor and bone material can be observed, as is the case for δ^18^O_CO3_ (Fig. 5, Table 2).

### Oxygen and Carbon Isotope Compositions of the Molars of *A*. *terrestris* (Intra-jaw Variations)

**Figure 6:**
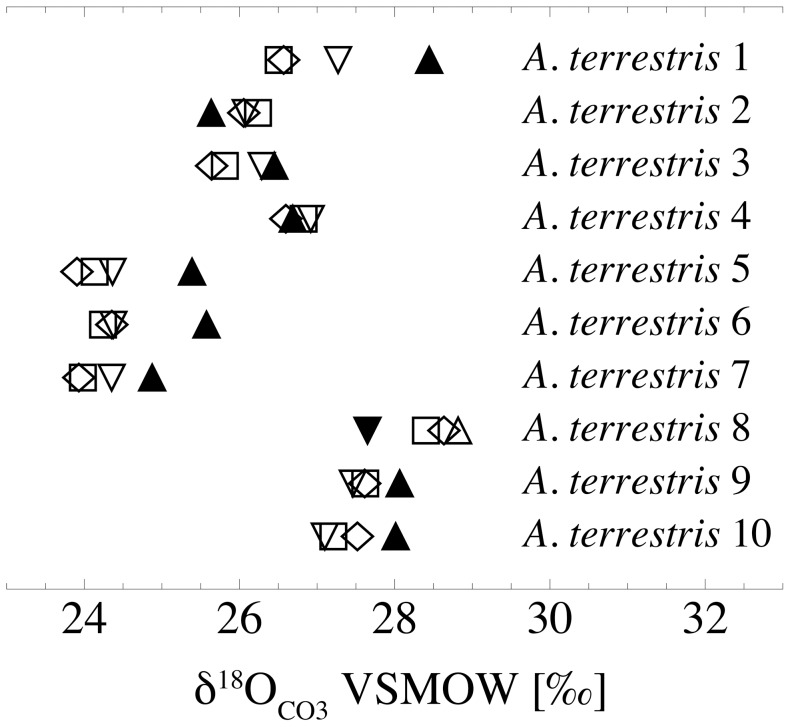
Intra-jaw variations in δ^18^O_CO3_ of *Arvicola terrestris*. Filled triangles = incisors, open diamonds = M1, open squares = M2, open down triangles = M3.

**Figure 7:**
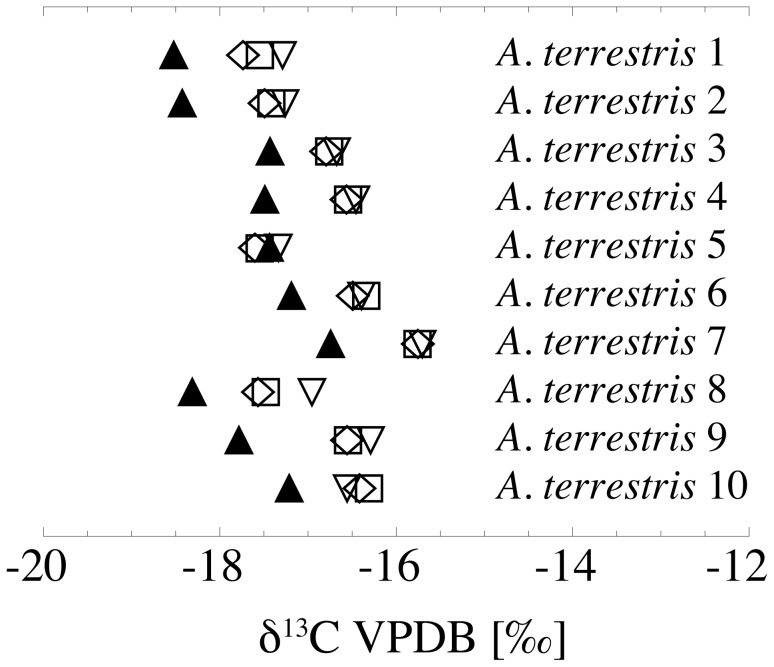
Intra-jaw variations in δ^13^C of *Arvicola terrestris*. Filled triangles = incisors, open diamonds = M1, open squares = M2, open down triangles = M3.

In *A*. *terrestris* the intra-jaw range (n = 10) between the molars (M1–M3) of the same individual is 0.2 to 1.0‰ in δ^18^O_CO3_ and 0.1 to 0.6‰ in δ^13^C. The overall intra-jaw range (I1, M1–M3) varies from 0.3 to 1.9‰ in δ^18^O_CO3_, while in most cases the incisors have higher values than the molars (Fig. 6, Table S1). In δ^13^C, nearly all incisors have lower values than the corresponding molars, with overall intra-jaw variations between 0.3 to 1.5‰ (Fig. 7, Table S1). Mean values for M1, M2, and M3 are 26.1±1.6‰, 26.1±1.5‰ and 26.2±1.3‰ in δ^18^O_CO3_ and −16.9±0.6‰, −16.8±0.6‰ and −16.7±0.5‰, respectively (n = 10 of each tooth type).

### Oxygen Isotope Analyses of the Phosphate in the Bioapatite (δ^18^O_PO4_) of *A. terrestris*

**Figure 8:**
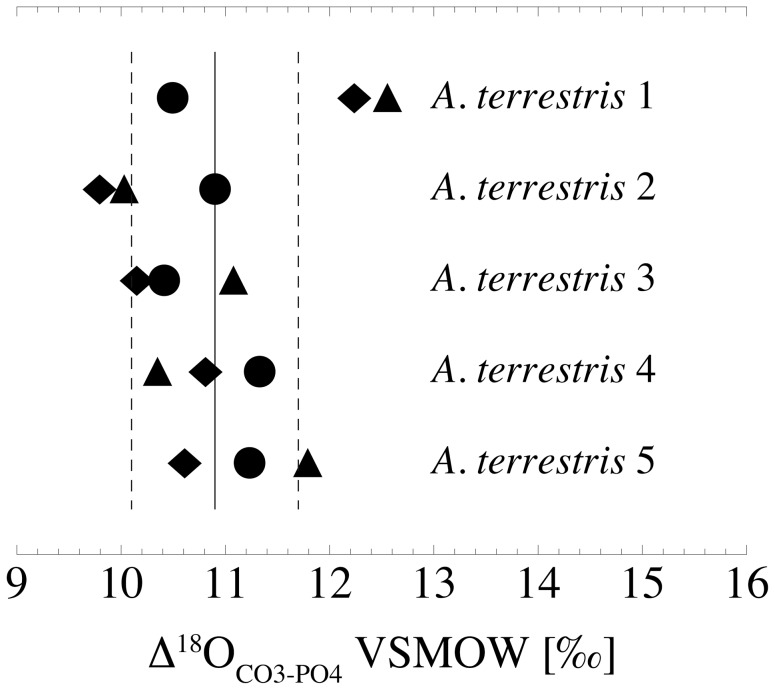
Δ^18^O _CO3-PO4_ of incisors, first molars and bone in *A*. *terrestris* 1 to 5. Triangles represent the incisors, diamonds the first molars and circles the bones. The average value for all samples is 10.9±0.8‰ (solid and dashed lines).

The mean δ^18^O_PO4_ of skeletal apatite from *A. terrestris* are 15.4±1.1‰ (bulk incisors, n = 5), 14.9±1.3‰ (bulk M1, n = 5) and 14.9±0.8‰ (bone, n = 5). The corresponding δ^18^O_CO3_ means are 26.5±1.2‰, 25.8±1.1‰ and 25.6±1.2‰, leading to an Δ^18^O_CO3-PO4_ of 11.2±1.0‰ (incisors), 10.9±0.4‰ (M1) and 10.7±0.9‰ (bone), respectively. The overall average Δ^18^O_CO3-PO4_ is 10.9±0.8‰ (Fig. 8, Table 3).

**Table 3:** δ^18^O_CO3_, δ^18^O_PO4_ and Δ^18^O _CO3-PO4_ of incisors, first molars and bone material of *A. terrestris* (n = 5).

Species	Material	δ^18^O_CO3_	δ^18^O_PO4_	Δ^18^O _CO3-PO4_
		(‰ vs. VSMOW)	(‰ vs. VSMOW)	(‰)
*Arvicola terrestris* 01	I^1^ sin.	28.4	15.9	12.5
*Arvicola terrestris* 01	M1 sin.	26.6	16.1	10.5
*Arvicola terrestris* 01	bone	27.7	15.4	12.3
*Arvicola terrestris* 02	I^1^ sin.	25.6	15.6	10.0
*Arvicola terrestris* 02	M1 sin.	26.1	15.2	10.9
*Arvicola terrestris* 02	bone	25.5	15.7	9.8
*Arvicola terrestris* 03	I^1^ sin.	26.4	15.4	11.0
*Arvicola terrestris* 03	M1 sin.	25.6	15.2	10.4
*Arvicola terrestris* 03	bone	25.2	15.0	10.2
*Arvicola terrestris* 04	I^1^ sin.	26.7	16.3	10.4
*Arvicola terrestris* 04	M1 sin.	26.6	15.3	11.3
*Arvicola terrestris* 04	bone	24.6	13.8	10.8
*Arvicola terrestris* 05	I^1^ sin.	25.4	13.6	11.8
*Arvicola terrestris* 05	M1 sin.	23.9	12.7	11.2
*Arvicola terrestris* 05	bone	25.2	14.6	10.6
**Mean (I1)**		**26.5±1.2**	**15.4±1.0**	**11.2±1.0**
**Mean (M1)**		**25.8±1.1**	**14.9±1.3**	**10.9±0.4**
**Mean (bone)**		**25.6±1.2**	**14.9±0.7**	**10.7±1.0**
**Overall mean**				**10.9±0.8**

### Minimum Sample Size Calculations

Minimum sample sizes, calculated with the bootstrapping approach used by Fox-Dobbs et al. [100] are in the same range or smaller than previously published data from large mammals (e.g., [28]–[30], [32]; Table 4). From all randomly chosen subsamples with n_sub_ ≥4, 95% or more range within 1σ of the mean of the complete data. In the case of a subsample size of n_sub_ ≥7, even a 99% confidence is reached for a range within 1σ of the mean of the whole dataset.

**Table 4:** Results of the bootstrap analysis of δ^18^O_CO3_ for rodent incisors (n = 10 in each species).

	Number of subsamples out of 1000 random replicates of n_sub_ within 1σ						
	Arvicolids (incisors)				Murids (incisors)		
n_sub_	*A*. *terrestris*	*M*. *glareolus*	*M*. *agrestis*	*M*. *arvalis*	*A*. *sylvaticus*	*M*. *musculus*	*R*. *norvegicus*
2	801	819	831	802	829	842	860
3	918	918	911	909	894	897	936
4	963	951	957	950	955	950	969
5	984	970	982	971	978	968	985
6	>990	981	990	983	986	982	>990
7 to 9	>990	>990	>990	>990	>990	>990	>990

## Discussion

### Inter- and Intra-specific Variations in δ^18^O_CO3_

Mean δ^18^O_CO3_ values of the analysed species (Fig. 3) show no, or only minor, differences. This suggests that probably all taxa drank from isotopically similar water sources. The higher δ^18^O_CO3_ in *R*. *norvegicus* compared to the other rodent species may be attributed to the juvenile status of the individuals and might reflect a remnant suckling signal.

Due to the lack of published small mammal δ^18^O_CO3_ data, the intra-population variability of the analysed species can only be compared to published δ^18^O_PO4_ values of rodents. The ranges from 2.0 to 3.9‰ (excluding one individual of *M. glareolus*, see results section) for the incisor teeth are much larger than the range in δ^18^O_PO4_ of 0.7 to 0.8‰ for fat dormice (*Glis glis*). However, only three specimens were investigated by Lindars et al. [83]. Bones of *Pitymus* sp., *M*. *arvalis* and *A*. *terrestris* had δ^18^O_PO4_ intra-population variations of 0.8 to 2.2‰, 0.4 ‰ and 1.3 to 2.3‰, respectively [36]. These ranges are lower than or reach the lower limit of the rodent δ^18^O_CO3_ variability in the present study. However, the lowest value of the aforementioned ranges is again based on three to four individuals only and derives mainly from mixed bone material.

Compared to large mammals, the observed variations of 2.0 to 3.9‰ are in the same range as previously reported δ^18^O_CO3_ values for North American bison (*Bison bison*) with 2.4 to 3.0‰ [29], Baird’s tapir (*Tapirus bairdii*) with 1.4 to 2.6‰ [101], feral and domestic horses (*Equus caballus*) with 2.0 to 6.5‰ [28], [30], [35], and bobcats (*Lynx rufus*) with 3.1‰ intra-population variability at a single locality [32]. Other published intra-population variabilities including three or more individuals for large terrestrial mammals are significantly larger than those observed in the present study, as is the case for mule deer (*Odocoileus hemionus*) with a range of 6.2‰, coyotes (*Canis latrans*) with 6.3‰ [32], domestic yaks (*Bos grunniens*) with 3.7 to 8.5‰ and domestic goats (*Capra aegagrus hircus*) with 5.4 to 10.6‰ intra-population variability [30].

### Inter- and Intra-specific Variations in δ^13^C

Mean δ^13^C values of the analysed species are in part significantly distinct from each other, notably between arvicolids and murids, indicating dietary differences (Fig. 3). The intra-population variations in δ^13^C have a wide range from 1.8 to 12.7‰, reflecting variations in the δ^13^C values of their diet and in the nutritional behaviour (i.e., dietary specialists vs. dietary opportunists). Previously published data on intra-population variations from large mammals range from 0.7 to 8.5‰ [29], [30], [32], [101].

The average carbon isotope fractionation between diet and bioapatite in rodents (determined on bone material) is+9.9‰ [1], [23], [102]. This suggests average diet δ^13^C values between −29.6 and −26.1‰ for the arvicolids. For the murids, the average diet δ^13^C values are higher: −25.8‰ in *A*. *sylvaticus*, −22.0‰ in *M*. *musculus* and −19.5‰ in *R*. *norvegicus*.

The low intra-population variations of *A*. *terrestris*, *M. glareolus* and *M*. *agrestis* (<2.2‰) indicate little dietary variations, which is supported for *A*. *terrestris and M*. *agrestis* by the observation of nearly exclusively herbivorous diets [103], [104]. An exclusively herbivorous diet is also known for *M. glareolus* in various regions [105]. The diet of *M*. *arvalis* and *A*. *sylvaticus* consists of a considerable but varying amount of invertebrates [106], [107] explaining the higher variability of 4.0 and 5.3‰, respectively. Mean values of these species correspond to a typical C_3_ diet. The highest variabilities in δ^13^C within the individuals from a single population were observed in *R*. *norvegicus* and *M*. *musculus*, with 10.2 and 12.7‰, respectively. Both are synanthropic species with a very opportunistic diet that commonly includes human food supplies and food waste [108]–[110]. Thus, the high variability in δ^13^C is likely caused by the presence or absence of C_4_-sugars and/or products containing tissues from animals fed on a C_4_ diet in the rodent diet.

### Variations in δ^18^O_CO3_ and δ^13^C between Incisor and Bone Material

The δ^18^O_CO3_ values of the incisors differ by up to ∼4‰ from that of bone material (Table S1), which can be explained by different time intervals in the mineralisation of both tissues. Rodent incisors (and also the molars of most arvicolids) are permanently growing and reflect a time span of a few weeks [86], [87], whereas no pronounced remodeling of bone tissue occurs in rodents. Thus, bones archive a nearly life-long record [88]. This may also explain the phenomenon of why nearly 90% of the analysed individuals have higher δ^18^O_CO3_ values in the incisors than in the bone material (Fig. 4). Taking into account the general population dynamics of small rodents in temperate regions with population maxima in late summer (see also materials and methods section), the incisor δ^18^O_CO3_ of a large number of individuals are likely biased towards the ^18^O-enriched precipitation of the warm summer months (Fig. 2). In contrast, bone material was formed over a longer time span, including the colder spring season or even the winter months in case of adult individuals that survived this period. This is further supported by the standard deviations of the bone-incisor offsets in each species (0.8 to 1.8‰), which reflects the different times of mineralisation of both tissues in relation to a variable age structure of the individual specimens. One exception is the juvenile *R*. *norvegicus* specimens that have a relatively uniform bone-incisor offset within the five analysed individuals (∼3‰), with a small standard deviation of only 0.3‰. This likely reflects their more or less identical age and hence similar time intervals recorded in bone and incisor δ^18^O_CO3_ values.

The δ^13^C values of the bulk incisor teeth, with two exceptions (Table S1), deviate less than 2‰ from the δ^13^C results of the respective bone material, and no general trend to a skewed variation, as is the case for δ^18^O_CO3_, can be observed (Fig. 5). These variations can be explained by variations in the carbon isotope composition of the consumed food during the different mineralisation times of incisor and bone material.

### Intra-jaw Variations in δ^18^O_CO3_ and δ^13^C of *A*. *terrestris*

The δ^18^O_CO3_ of the molars of *A*. *terrestris* deviates from the incisor oxygen isotope composition of the same individual between 0.3 and 1.9‰, indicating slight differences in the time periods of mineralisation of both tooth types. For other arvicolids (*Microtus*) a complete renewal of incisors within four to seven weeks is reported [87], whereas first and second molars of *Microtus* underly a complete renewal in a time interval of eight to twelve weeks [86]. Considering this, as well as the fact that the molars mostly have lower δ^18^O_CO3_ values than incisors (Table S1; analogous to the incisor-bone offset), a small bias by seasonally driven population dynamics is likely the cause for the inter-tooth offset.

The difference in δ^13^C between molar teeth and incisors of all analysed individuals is less than 2‰, comparable to the bone-incisor variations and reflecting varying carbon isotope compositions of the ingested food (Table S1). Given that the owl pellets represent a seasonally biased taphocoenosis, the lower δ^13^C values in nearly all incisors compared to the molars of the same individuals can result either from a seasonal change in availability of food plants or from seasonal changes in the carbon isotope composition of the same plant species, which can be in the range of several per mil within one plant species (e.g., [67], [111]).

Variations among M1 to M3 of the same animal are generally lower, both in δ^18^O_CO3_ (0.2 to 1‰) as well as in δ^13^C (0.1 to 0.6‰), indicating a more or less contemporaneous growth and mineralisation of these molars.

### The Δ^18^O_CO3-PO4_ in *A*. *terrestris*

Comparing incisors, molars and bone, the differences between δ^18^O_CO3_ and δ^18^O_PO4_ of each element overlap within error (Table 3). The overall Δ^18^O_CO3-PO4_ of 10.9±0.8‰ is high and at the upper limit of previously reported values for large and medium-sized mammals (7.5 to 10.6‰; [43]–[45], [47], [49], [112]) that include perissodactyls, artiodactyls and carnivorans. The only reported data for rodents (laboratory rats, *Rattus norvegicus*) from two different settings show a highly variable Δ^18^O_CO3-PO4_ of 8.5 and 11.4‰, respectively [48]. Further studies are needed to evaluate a possible species or body temperature dependence as suggested by Martin et al. [45] or a connection to seasonal variations in the δ^18^O of drinking water and/or a food source of phosphate as supposed by Kirsanow and Tuross [48].

### Minimum Sample Size Calculations

The bootstrapping results of our δ^18^O_CO3_ data from rodents (more than 95% of the subsamples are within ±1σ of the mean if n_sub_ ≥4, and even more than 99% if n_sub_ ≥7) agree to the minimum sample size recommendations for most large mammals given in previous studies [28], [29], [32] as well as for the horses from the study of Wang et al. [30]. For goats and yaks, a considerably higher minimum sample size for reliable estimates of the population mean δ^18^O within a 95% confidence level has been determined [30], presumably related to differences in physiology and/or a different nutritional or rather drinking behaviour. Our data for rodents indicate that the minimum sample size recommendations, determined for large mammals, potentially can be extended to small mammal species (i.e., rodents). Further taxon-specific studies, however, are needed to confirm if the present finding is valid for small mammals in general.

### Suitability for the δ^18^O Reconstruction of Local Water

The δ^18^O_CO3_ variability of different sympatric rodent taxa from the studied locality is low. This suggests the reliability of a δ^18^O_CO3/PO4_-δ^18^O_LW_ calibration equation based on a generic, family or even higher taxonomic level. However, factors that can affect the oxygen isotope composition of biogenic apatite (e.g. habitat, drinking behaviour, population dynamics, body size and thermophysiology) should be considered carefully before inferring drinking water δ^18^O values from existing calibration equations or the development of new ones.

**Figure 9:**
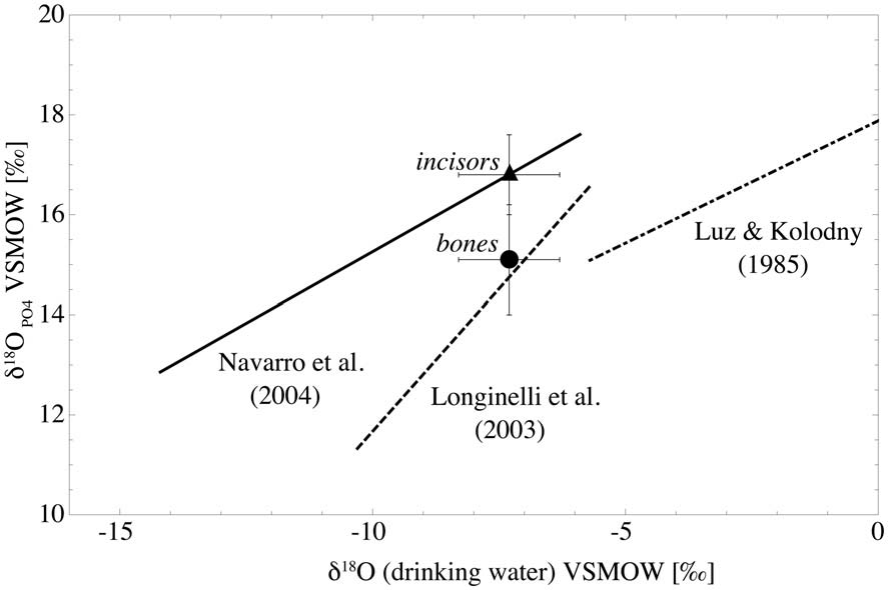
Tooth (triangle) and bone (circle) mean values of all analysed samples converted from δ^18^O_CO3_ to δ^18^O_PO4_ and plotted versus the long term δ^18^O average of local precipitation (-7.3‰). Dashed lines show the existing δ^18^O_PO4_-δ^18^O_LW_ regression lines for rodents developed mainly on bone material by Luz & Kolodny (1985) and Longinelli et al. (2003) [4], [36]. The solid line shows the δ^18^O_PO4_-δ^18^O_LW_ calibration equation from Navarro et al. (2004) [16], which is mainly based on tooth material.

To assess if calibration equations developed on bone material can be unrestrictedly applied to tooth data and vice versa, incisor and bone mean values were compared to each of the three existing δ^18^O_PO4_-δ^18^O_LW_ calibration equations for different rodent taxa (Table 5). The δ^18^O_PO4_ values were calculated from the measured δ^18^O_CO3_ values, applying the Δ^18^O_CO3-PO4_ of 10.9±0.8‰ determined for *Arvicola terrestris*. Thus, combining the data of all seven analysed species, the calculated mean δ^18^O_PO4_ values are 16.8±0.8‰ (n = 70) for incisor teeth and 15.1±1.1‰ (n = 35) for bones. These values were plotted versus the long-term local mean δ^18^O_LW_ of −7.3‰ in relation to existing δ^18^O_PO4_-δ^18^O_LW_ regression lines for rodents [4], [16], [36] (Fig. 9).

**Table 5:** Existing calibration equations between δ^18^O_LW_ and δ^18^O_PO4_ for rodents.

Study	developed calibration equation	skeletal tissue used	included genera
Luz and Kolodny (1985)	δ^18^O_LW_ = (δ^18^O_PO4_-17.88)/0.49	bone	*Rattus*
Longinelli et al. (2003)	δ^18^O_LW_ = (δ^18^O_PO4_-23.07)/1.14	bone (nearly solely)	*Apodemus*, *Pitymus*, *Arvicola*, *Microtus*
Navarro et al. (2004)	δ^18^O_LW_ = (δ^18^O_PO4_-20.98)/0.572	molars, incisors	*Microtus*, *Myodes*, *Lemmus*

The regression line of Luz and Kolodny [4] is based on bone material from laboratory rats, and that of Longinelli et al. [36] is based nearly exclusively on bones of wild murids and arvicolids (including the genera *Apodemus*, *Arvicola*, *Microtus* and *Pitymus*). The regression of Navarro et al. [16] was primarily derived using molar and incisor teeth from wild small arvicolids of the genera *Myodes* and *Microtus*.

As the calibration equation of Luz & Kolodny [4] covers only a δ^18^O_LW_ range outside the δ^18^O of local precipitation in our sample area, a direct comparison to our data is precarious. However, extrapolation of the regression line reveals that the bone-derived δ^18^O values from the present study are much closer to this line than the tooth values (Fig. 9).

A much better fit is obtained if the bone-based equation of Longinelli et al. [36] is compared with our bone data, which directly plot on the respective calibration line. A similar concordance exists by comparing our tooth-data with the tooth-based equation of Navarro et al. [16] (Fig. 9).

This indicates that the choice of the skeletal element used for the calibration between the δ^18^O of biogenic apatite and δ^18^O_LW_ has an important influence, which can be assigned to different mineralisation periods and seasonally driven population dynamics in rodents as discussed above. In the case of the study by Luz and Kolodny [4], an additional explanation for differences from our data could be the fact that the respective equation is based on laboratory animals that often differ in their physiology from field populations, which can have an impact on the oxygen isotope composition of their biogenic apatite (e.g., [82]). Furthermore, as laboratory rats were raised under controlled conditions with a constant diet and water source, this precludes the effect of any seasonal bias.

Previous studies have shown that tooth enamel should be the material of choice for oxygen isotope studies in fossil mammals, because it is less prone to diagenetic alteration than dentine or bone material [6], [113]. Therefore, future δ^18^O_PO4_-δ^18^O_LW_ calibration equations of modern taxa that aim to reconstruct palaeoenvironmental conditions should be based on tooth material instead of bone. Furthermore, the same tooth type available also from the fossil taxa should be used, in order to avoid seasonal bias due to different tooth formation patterns.

### Conclusions

The observed inter-specific variations in δ^18^O_CO3_ within arvicolid and murid incisors are low. Intra-specific variations in the δ^18^O of the investigated rodent bioapatite samples are in the same range as most previously observed values from large mammals. Statistical evaluations show that minimum sample size recommendations from existing studies on large mammals agree well with the results from the present study, indicating at least 95% confidence that from a subsample size of ≥4 mean values range within 1σ of the mean of the sample population. The observed minor differences in the intra-specific δ^18^O variability of rodents compared to large and medium-sized mammals corroborate the applicability of rodents in palaeoclimatic studies. Due to the differing time intervals recorded in the different skeletal tissues (incisors, molars, bone), rodents can show considerable variations in δ^18^O within a single individual, caused by seasonal biased δ^18^O_LW_ ingested by individuals. This underscores the importance of using analogous tissue material when δ^18^O data from fossil rodents are evaluated with δ^18^O_PO4_-δ^18^O_LW_ calibration equations based on modern taxa. Comparisons of the δ^18^O data from the analysed arvicolids and murids with the existing calibration equations relative to the δ^18^O data of local precipitation shows that a significant deviation from the expected value occurs when bone-based equations are applied to tooth-data and vice versa. The Δ^18^O_CO3-PO4_ of 10.9±0.8‰ determined for *A. terrestris* is at the upper limit of data from large mammals and ranges within the two previously reported values from another rodent species (*R*. *norvegicus*). Within the present study, no significant disadvantages in the interpretation of δ^18^O data from rodent bioapatite compared to large- and medium-sized mammals were observed. Nevertheless, it has to be taken into account that fossil rodent taphocoenoses in temperate regions mostly underlie seasonal and spatial predator-prey population dynamics.

The δ^13^C data mirror the different nutritional strategies of the analysed species (i.e., dietary specialisation vs. dietary opportunism). The range of variations within a single species can vary considerably but agrees well with field observations of the respective nutritional behaviour. Thus, multiple analyses from different individuals are an important prerequisite to get deeper insight into specific nutritional adaptions from fossil small mammals. Seasonally driven variations in the carbon isotope composition of the ingested food are reflected in different skeletal tissues, caused by differing time intervals of incisor, molar and bone mineralisation.

The present study underscores the high potential of stable isotope signatures of fossil small mammal skeletal remains to reconstruct past environmental and climatic conditions. However, further detailed systematic investigations of modern analogous communities of small mammals are necessary for a refined understanding of the incorporation of oxygen and carbon isotope compositions in the bioapatite of their teeth and bones and to explore the full potential of fossil rodents for palaeoenvironmental research.

## Supporting Information

Table S1 Detailed summary of analytical results
(XLS)
